# Sex difference in thermal preference of adult mice does not depend on presence of the gonads

**DOI:** 10.1186/s13293-017-0145-7

**Published:** 2017-07-11

**Authors:** Kasiphak Kaikaew, Jacobie Steenbergen, Axel P. N. Themmen, Jenny A. Visser, Aldo Grefhorst

**Affiliations:** 1 Department of Internal Medicine, Erasmus MC, University Medical Center Rotterdam, PO Box 2040, 3000 CA Rotterdam, The Netherlands; 2 Department of Physiology, Faculty of Medicine, Chulalongkorn University, 1873, Rama IV Road, Pathumwan, Bangkok 10330 Thailand

**Keywords:** Sex, Gonadectomy, Estrogen, Progesterone, Testosterone, Thermosensing, Body temperature regulation, Thermogenesis, Nesting behavior, Dark-light cycle

## Abstract

**Background:**

The thermoneutral zone (TNZ) is a species-specific range of ambient temperature (*T*_a_), at which mammals can maintain a constant body temperature with the lowest metabolic rate. The TNZ for an adult mouse is between 26 and 34 °C. Interestingly, female mice prefer a higher *T*_a_ than male mice although the underlying mechanism for this sex difference is unknown. Here, we tested whether gonadal hormones are dominant factors controlling temperature preference in male and female mice.

**Methods:**

We performed a temperature preference test in which 10-week-old gonadectomized and sham-operated male and female C57BL/6J mice were allowed to choose to reside at the thermoneutral cage of 29 °C or an experimental cage of 26, 29, or 32 °C.

**Results:**

All mice preferred a *T*_a_ higher than 26 °C, especially in the inactive phase. Choosing between 29 and 32 °C, female mice resided more at 32 °C while male mice had no preference between the temperatures. Hence, the preferred *T*_a_ for female mice was significantly higher (0.9 ± 0.2 °C) than that for male mice. However, gonadectomy did not influence the *T*_a_ preference.

**Conclusions:**

Female mice prefer a warmer environment than male mice, a difference not affected by gonadectomy. This suggests that thermal-sensing mechanisms may be influenced by sex-specific pathways other than gonadal factors or that the thermoregulatory set point has already been determined prior to puberty.

**Electronic supplementary material:**

The online version of this article (doi:10.1186/s13293-017-0145-7) contains supplementary material, which is available to authorized users.

## Background

Mammals prefer ranges of ambient temperature (*T*_a_) within their species-specific thermoneutral zone (TNZ) at which their metabolic rate is at its lowest and theoretically equal to their basal metabolic rate. To control the core body temperature (*T*_c_) in TNZ, only dry heat loss, i.e., skin blood flow, is sufficient. However, metabolic heat production or evaporative heat loss is required to regulate *T*_c_ at a *T*_a_ outside the TNZ [1]. Disturbances in *T*_a_ can alter normal physiological responses such as dietary requirement, cardiovascular functions, and reproduction capacity [2, 3]; thus, it is important to be aware of the changes of *T*_a_. The TNZ for an adult mouse is considered to be broad and in between 26 and 34 °C, and mice therefore prefer a *T*_a_ between 29 and 31 °C [4]. However, general animal facilities apply a *T*_a_ for laboratory mice in the range of 20–24 °C [5], which matches a thermal comfort of husbandry staffs and handling procedures but poses a persistent moderate cold stress to mice [6] affecting their physiological processes [7].

Many factors contribute to distinct patterns of thermal preference among mice. For instance, since mice are nocturnal animals, their motor activity has a diurnal rhythm which peaks in the dark phase. An increase in heat production related to this physical activity during the active phase results in a preference for a lower *T*_a_ to maintain *T*_c_ at night [6]. In addition, a higher *T*_a_ is required by neonatal mice due to limitations in their thermal regulatory mechanisms [8]. Elderly mice also require higher *T*_a_ due to changes in total body surface area (BSA). Upon growth, the BSA to body mass ratio decreases resulting in less capacity to exchange heat with the environment [4, 9]. Finally, thermal differences in mice have a sex-dependent pattern: female mice prefer a slightly higher *T*_a_ than male mice of the same age, especially in the inactive phase [10].

The mechanism(s) that underlie the differences in thermal regulation and preference between sexes are not clearly understood, but gonadal hormones such as the female sex hormones estradiol and progesterone and the male sex hormone testosterone might be considered dominant factors. The overall *T*_c_ of post-pubertal female mice is 0.2–0.5 °C higher than that of male mice, especially when the levels of progesterone and, to a lesser extent, estradiol are elevated during the estrous cycle [11]. Of interest, this higher *T*_c_ in female mice is not due to the difference in locomotor activity between male and female mice [11]. Progesterone shifts the thermoregulatory set point in the central nervous system resulting in an increase of basal *T*_c_ in the luteal phase of the human ovarian cycle [12]. Since the mouse estrous cycle lasts only 4–5 days and does not include a true luteal phase, major and prolonged elevations of progesterone are only seen during (pseudo)pregnancy [13]. Estradiol influences energy balance by increasing the energy expenditure through modulation of central neuronal circuits [14], which includes the production of heat by brown adipose tissue (BAT) [15]. BAT is an important source of heat production in rodents, especially in the inactive phase. Interestingly, both estradiol and progesterone also directly stimulate biogenesis of brown adipocytes while testosterone inhibits differentiation of brown adipocytes [16, 17]. However, the effects of gonadal hormones on the thermal preferences in mammals are not clearly studied.

To elucidate the effects of gonadal hormones on thermal preference, we performed temperature preference tests (TPTs) in adult mice of both sexes, in a range of *T*_a_ close to their TNZ. The results of the TPTs will reveal whether removal of gonadal hormones by gonadectomy (GDX) affects temperature preference.

## Methods

### Animals and housing conditions

Six-week-old C57BL/6J mice (16 male and 16 female mice) were obtained from Charles River Laboratories (Maastricht, the Netherlands). Upon arrival, the mice were housed for 1 week in groups of three animals in standard laboratory cages (Sealsafe 1145T, Tecniplast, Buguggiate, Italy; 36.9 cm *L* × 15.6 cm *W* × 13.2 cm *H*) with bedding material (Lignocel BK 8/15, J. Rettenmaier & Söhne GmbH, Rosenberg, Germany) and nesting material (facial tissue paper, Tork, SCA Hygiene Products, Zeist, the Netherlands) on a 12:12-h light-dark cycle (lights on at 8 a.m.), at temperatures of 21–23 °C with 40–70% relative humidity. Chow food pellets (801727 CRM (P), Special Diets Services, Essex, UK) and water were available ad libitum. After the 1-week acclimatization, the mice were housed individually in a cage of the same setting for 3 days before the first series of TPTs was started. Thus, the mice were about 8 weeks old when enrolled in the experimental setups. All experimental procedures were performed with the approval of the Animal Ethics Committee at Erasmus MC, Rotterdam, the Netherlands.

### Temperature preference test setup

**Fig. 1:**
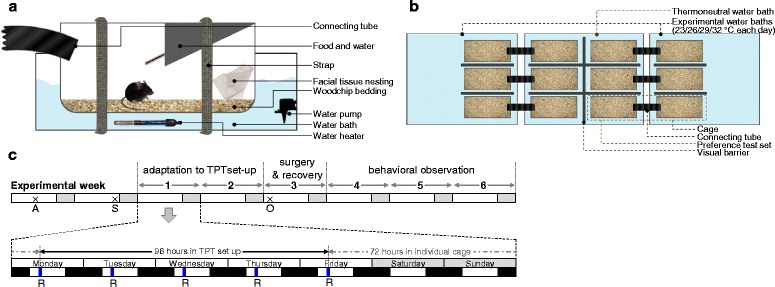
Experimental design. **a** Side view of each experimental cage showing cage enrichments provided for each mouse. **b** Top view of six temperature preference sets of which the two outer water baths were set at a different experimental temperature each day, while the middle water bath was kept constant at 29 °C. **c** Experimental scheme. Abbreviations: *A* arrival of mice, *S* separation to individual cage, *O* surgical operation, *R* daily routine care

For the TPT, a setup was designed based on the thermal preference apparatus by Gaskill BN et al. [18]. To connect two cages, an opening was drilled in one wall of each cage (4 cm diameter at the position of 7.5 cm from the cage floor) and a black corrugated connecting tube was placed, as shown in Fig. 1a, b. Water baths, in which electric submersible aquarium heaters and water pumps (Superfish Combi Heater and Aqua-Flow, Aquadistri, Cambridgeshire, UK) were installed, were used to maintain a constant *T*_a_ in the cages. The water bath was filled with water to the level of 7.5 cm depth, and the cages were fastened by fabric straps to ensure that the water level was 5 cm from the cage bottom. At this water level, the *T*_a_ inside the cage (measured at the floor) was less than 0.5 °C different from the temperature in the water bath. Each cage contained a bottle of water, food pellets, nesting material (one sheet of the facial tissue paper, two layers per sheet, size 20 cm × 21 cm, weight 1.3 g), and bedding material (0.5 cm depth of Lignocel BK 8/15 woodchips). The mice were allowed to freely explore both cages. To prevent position bias from adjacent cages, opaque plastic sheets were placed as visual barriers between the cages. In total, the setup consisted of six TPT sets (Fig. 1b) for studying six mice simultaneously.

### Experimental design

The total experimental procedure had a duration of 6 weeks, consisting of weeks 1 and 2 for adaptation to the TPT setup, week 3 for surgical procedure and recovery, and weeks 4 to 6 for behavioral observation (Fig. 1c). In the adaptation period (weeks 1 and 2) and the experimental period (weeks 4, 5, and 6), we performed the TPT in a total of 96 h after which the mice returned to their individual cages for the weekends. On Mondays, the temperature of the outer cages (the experimental cages) was set at 23 °C to mimic the general animal facility environment, while the temperature of the middle cages (the thermoneutral (TMN) cages) was set at 29 °C. The routine care was performed daily between 1 and 2 p.m. including replacing of bedding and nesting material, weighing mice and food, and adjusting the temperature of the experimental cages to 26, 29, and 32 °C on Tuesday, Wednesday, and Thursday, respectively. The temperature of the TMN cages was kept at 29 °C throughout the week. After the daily routine, the mouse was put back in its experimental cage. The cages and water baths were cleaned thoroughly at the end of every week. For the surgical week (experimental week 3), the mice were randomized to undergo GDX or sham operation on Monday. After the surgery, the mice were allowed to recover for 7 days in their individual cages. To reduce the effect of position bias, the mice were relocated to an adjacent TPT set in the following week.

### Gonadectomy

GDX was operated under isoflurane (Isofluraan, Teva Pharmachemie, Haarlem, the Netherlands) anesthesia and carprofen (Rimadyl Cattle, Pfizer, Capelle a/d IJssel, the Netherlands) analgesia. For the GDX of female mice, small incisions were made in both flanks to remove the ovaries. In the male mice, small incisions were made in the lower abdomen through which the testes were removed. Sham-operated animals underwent the same procedures without removal of ovaries or testes. Bleeding was verified and stopped and then the muscle layer and skin were sutured.

### Termination of mice

In the week after the last TPT, the mice were euthanized by cardiac puncture under isoflurane anesthesia. For the female mice, the uterus was dissected and weighed to verify if the ovariectomy was successful.

### Behavioral observation, data collection, and analysis

The thermal preference of each mouse was recorded by webcams (Exis, Trust, Dordrecht, the Netherlands) with the time-lapse function of Netcam Studio software (Moonware Studios) resulting in pictures with a 5-min interval that were collected for 23 h, excluding the hour of the daily routine period. In the dark period, the photographs were taken under red lights. After a pilot study (results not shown), the periods of 9–12 p.m. and 9–12 a.m. (starting 1 h after light off and on, respectively) were selected for the study. Data from the mice that failed to explore both the experimental and the TMN cage for the whole experimental period were excluded from data analysis.

After weighing the mice and the food in all the cages during the routine care, the bedding and nesting materials were gathered for further processing. Feces were sorted out from the woodchip bedding and weighed. The nesting material was unfolded, put on a black board, and photographed for analysis by three assessors. The “paperwork score,” modified from Kalueff et al. [19], was assessed to quantify how active a mouse was in biting and shredding the paper. This score ranges from 1 to 4: 1—without or little paper damage (<5% of destruction compared to the total area of the nest), 2—mild paper damage (5–25%), 3—moderate paper damage (25–50%), and 4—severe paper damage (>50%), as shown in Additional file 1: Figure S1. An average overall agreement of the paperwork score by three blinded assessors equaled to 84.7% (*κ* = 0.79), without any more-than-1 ordinal score discrepancy. Since the nesting material can be transferred from one cage to the other, we also assessed the “nest score” for each cage. This score is calculated as follows:

Nest score = ((paperwork score)/4) × (relative amount of nest in the cage).

In this, the relative amount of nest is a proportion of nest weight in that cage to the total weight of the nesting material provided to the animal, ranging from 0 to 1. For comparison between the experimental and the TMN cage of each mouse, the higher paperwork score and the higher nest score were used for data analysis.

The BSA of the mice was calculated by Meeh’s formula with the C57BL/6J specific constant as described by Cheung et al. [20]:

$$ \mathrm{B}\mathrm{S}\mathrm{A}=9.822\times {\mathrm{BW}}^{0.667}. $$

In this, BW is the body weight in gram and BSA is the surface in square centimeter (cm^2^).

Baseline characteristics of mice taken during the adaptation weeks are provided in Additional file 1: Table S1.

### Statistical analysis

The statistical tests were performed using IBM SPSS Statistics for Windows, version 21 (IBM Corp.) with *p* < .05 considered statistically significant. Unless otherwise stated, the effects of experimental *T*_a_, sex of mice, and gonadal status were analyzed by repeated three-way ANOVA, and then, the post hoc tests were evaluated by two-way ANOVA for the effects of sex and gonadal status in each *T*_a_ and Tukey tests to determine differences among groups. The *p* values of *T*_a_, sex, and gonadal status are indicated by *p*_T_, *p*_S_, and *p*_G_, respectively. The proportion of time that a mouse spent in each cage was arcsine transformed. The preference to either the TMN cage or the experimental cage in each *T*_a_ was analyzed by one-sample *t* test. Correlations between the time-weighted average *T*_a_ and other factors were calculated by Pearson’s correlation coefficient. All data are presented as mean ± SD unless otherwise indicated.

## Results

### Gonadectomy affected the energy balance pattern mainly in female mice

**Fig. 2:**
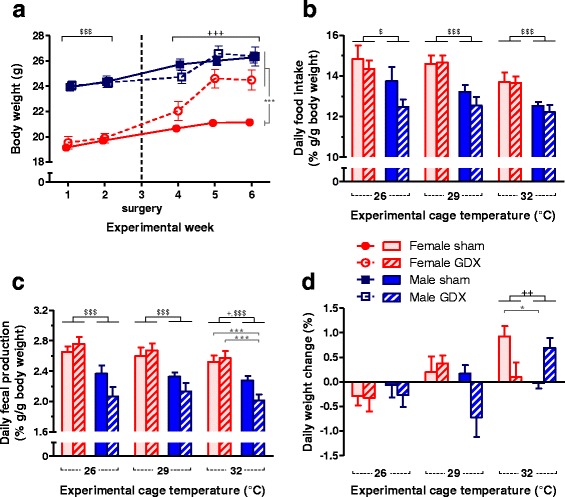
Gonadectomy affected the energy balance pattern mainly in female mice. **a** Gonadectomy increased body weight only in female mice. Weights of mice measured at the first day of each experimental week are plotted. **b** Daily food intake and **c** fecal production were higher in female mice with negative correlations with the experimental *T*_a_. **d** Body weight was unchanged during the 24-h period in the experimental setup when the experimental *T*_a_ was set at 26 and 29 °C, but some groups of mice gained weight when the experimental *T*_a_ was set at 32 °C. Two-way ANOVA: ^$^ *p*_S_ < .05, ^$$$^ *p*_S_ < .001, ^+^ *p*_S×G_ < .05, ^++^ *p*_S×G_ < .01, ^+++^ *p*_S×G_ < .001, and Tukey test: *p < 0.05, ***p < 0.001. *Error bar* indicates SEM, *n* = 7-8 per group

GDX differentially affected body weight (BW) in male and female mice. While ovariectomy increased BW of female mice, orchiectomy did not affect BW of male mice during the study period. As a result, the BW of gonadectomized female mice was higher than the sham-operated female mice in the last experimental week (two-way ANOVA: *p*_S_ < .001, *p*_G_ = .011, *p*_S×G_ = .014; Fig. 2a). In addition, the ratio of BSA to BW at the start of the last experimental week depended significantly on both sex and gonadal status (female sham 3.57 ± 0.03 cm^2^/g, female GDX 3.43 ± 0.09 cm^2^/g, male sham 3.32 ± 0.04 cm^2^/g, and male GDX 3.33 ± 0.07 cm^2^/g; two-way ANOVA: *p*_S_ < .001, *p*_G_ = .013, *p*_S×G_ = .006; Tukey post hoc test revealed *p* < .05 for all pairs except sham-operated male vs gonadectomized male mice).

In general, the experimental *T*_a_ influenced the relative daily food intake (*p*_T_ = .003) and female mice ate relatively more than male mice without an effect of gonadal status (*p*_S_ < .001; Fig. 2b). However, the negative correlation of *T*_a_ on daily food intake was only significant for sham-operated male mice (*r* = −.54, *p* = .011). In line with the food intake, the experimental *T*_a_ was related to the relative fecal production (*p*_T_ = .018). Moreover, the fecal production of female mice was higher than that of male mice, with a significant interaction between sex and gonadal status (*p*_S_ < .001, *p*_S×G_ = .049; Fig. 2c). Although the general trend of daily food intake (energy input) was negatively correlated with the experimental *T*_a_, the daily BW change of mice tended to positively correlate with the experimental *T*_a_ without a significant effect of sex or gonadal status but with a significant interaction between *T*_a_, sex, and gonadal status (*p*_T_ = .002, *p*_T×S×G_ = .002; Fig. 2d). The positive correlations between *T*_a_ and daily BW change were significant in sham-operated female mice (*r* = .62, *p* = .003) and gonadectomized male mice (*r* = .54, *p* = .007).

### Female mice preferred a higher ambient temperature, especially in the inactive phase

**Fig. 3:**
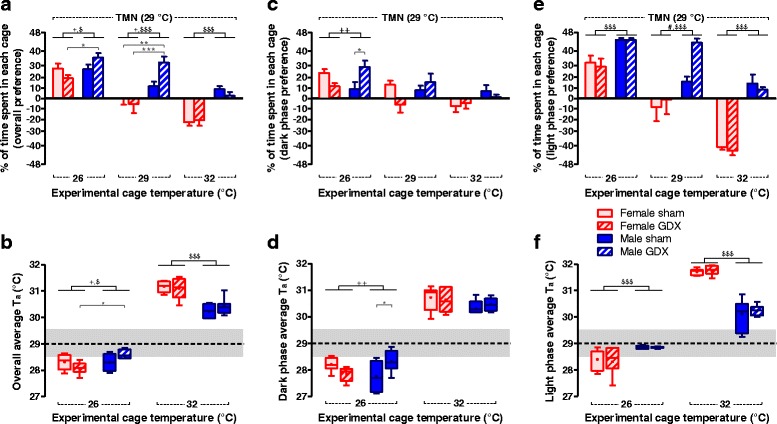
Female mice preferred a higher ambient temperature, especially in the inactive phase. **a** Both male and female mice preferred the TMN over the lower *T*_a_ (26 °C) while female but not male mice preferred the higher *T*_a_ (32 °C) over TMN. The thermal preference in the **c** dark (active) phase and **e** light (inactive) phase showed distinct patterns. The percentage of time that mice spent in each cage is represented on the *y*-axis as an angular transformed scale. Zero means the mice equally spent time in both cages. A positive value means preference to the TMN cage while a negative value means a preference to the experimental cage, of which the temperature is indicated on the *x*-axis. **b**, **d**, **f** *Box-and-whisker plots* (box indicates interquartile range and whisker is plotted using Tukey method) show the time-weighted average *T*_a_ that the mice were exposed to. The *gray area* indicates a temperature range of 28.5–29.5 °C. Two-way ANOVA: ^$^ *p*_S_ < .05, ^$$$^ *p*_S_ < .001, ^#^ *p*_G_ < .05, ^+^ *p*_S×G_ < .05, ^++^ *p*_S×G_ < .01, and Tukey test: *p < .05, **p < .01, ***p < .001. *Error bar* indicates SEM, *n* = 7–8 per group

The time mice spent in the TMN cage was influenced by experimental *T*_a_, sex, and the interaction between these factors, but was not altered by gonadal status (*p*_T_ < .001, *p*_S_ < .001, *p*_T×S_ = .002, *p*_T×S×G_ = .004; Fig. 3a). When the *T*_a_ of the experimental cages was set at 26 °C, all groups of mice preferred to reside in the TMN cages rather than the experimental cages (*p*_S_ = .038, *p*_S×G_ = .026; Fig. 3a). Calculated from the cage preference data, the time-weighted average *T*_a_ the mice were exposed to was slightly higher for male than for female mice, with a significant interaction between sex and gonadal status (female sham 28.3 ± 0.3 °C, female GDX 28.1 ± 0.2 °C, male sham 28.3 ± 0.3 °C, male GDX 28.5 ± 0.3 °C; *p*_S_ = .044, *p*_S×G_ = .030; Fig. 3b). When the *T*_a_ of the experimental cages was set the same as of the TMN cages at 29 °C, male mice (both sham-operated and gonadectomized) resided more in the TMN cages while female mice had no preference to either cage (*p*_S_ < .001, *p*_S×G_ = .033; Fig. 3a). When the *T*_a_ of the experimental cages was set at 32 °C, female mice (both sham-operated and gonadectomized) spent more time in the experimental cage than in the TMN cage, while sham-operated male mice preferred the TMN cage and gonadectomized male mice had no preference for either the TMN or the 32 °C cage (*p*_S_ < .001; Fig. 3a). Calculated from the cage preference data, the time-weighted average *T*_a_ the mice were exposed to when able to choose between 29 and 32 °C was significantly higher for female mice than male mice, without an effect of gonadal status (female sham 31.2 ± 0.2 °C, female GDX 31.1 ± 0.4 °C, male sham 30.2 ± 0.2 °C, male GDX 30.4 ± 0.3 °C; *p*_S_ < .001; Fig. 3b). Thus, in general, the female mice preferred a higher *T*_a_ than male mice, a difference that was not affected by presence of the gonads.

Next, we determined whether *T*_a_ preference between the sexes was different in the active or inactive phase and found that the sex of mice influenced the thermal demand differently between the dark (active) phase (*p*_T_ < .001, *p*_T×S×G_ = .002; Fig. 3c) and the light (inactive) phase (*p*_T_ < .001, *p*_S_ < .001, *p*_T×S_ = .002, *p*_T×G_ = .013; Fig. 3e). When the experimental *T*_a_ was set at 26 °C, sham-operated female, gonadectomized female, and gonadectomized male mice preferred to stay in the TMN cage in the dark phase, while sham-operated male mice had no preference to either cage in the dark phase. In the light phase, all groups preferred the TMN cage over the experimental 26 °C cage with a more prominent preference in male mice, without an effect of gonadal status. When the experimental *T*_a_ was set at 32 °C, all groups equally resided in both the 32 °C and the TMN cages in the dark phase. In the light phase, sham-operated and gonadectomized female mice preferred the 32 °C cage over the TMN cage, whereas gonadectomized male mice preferred the TMN cage and sham-operated male mice showed no preference to either cage.

It is thus evident that GDX did not alter the sex-dependent thermal preferences when analyzing the active and inactive phase independently, except only for the male mice in the dark phase at experimental *T*_a_ of 26 °C (*p* = .030). In addition, the time-weighted average *T*_a_, calculated from the location where the mice resided, was different between the active and inactive phases (Fig. 3d, f, respectively). The difference between male and female mice was the most obvious when they were able to choose between 29 and 32 °C in the inactive phase: female mice preferred higher *T*_a_ than male mice without an effect of gonadal status (female sham 31.7 ± 0.1 °C, female GDX 31.8 ± 0.2 °C, male sham 30.1 ± 0.6 °C, male GDX 30.2 ± 0.2 °C; *p*_S_ < .001; Fig. 3f).

### The location of activities was affected mainly by ambient temperature and partially by orchiectomy

**Fig. 4:**
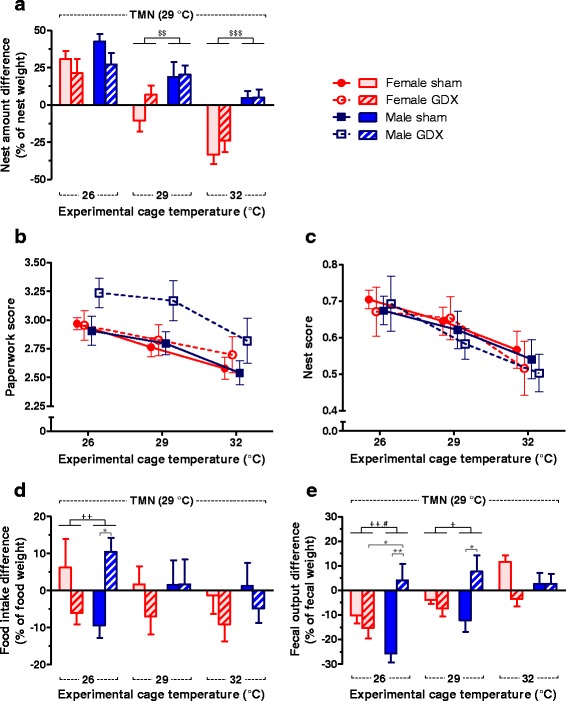
The location of activities was affected mainly by ambient temperature and partially by orchiectomy. **a** At the experimental *T*_a_ of 26 °C, all groups transferred the nest to the TMN cage, whereas at the experimental *T*_a_ of 32 °C, only female mice transferred the nest to the experimental cage. The difference of nest amount found in each cage is represented on the *y*-axis. Zero means the mice did not transfer the nesting material. A positive value means transfer of nesting material to the TMN cage while a negative value means transfer to the experimental cage, of which the *T*_a_ is indicated on the *x*-axis. Nest-relating activities revealed negative correlations of *T*_a_ to the **b** paperwork score and **c** nest score without an effect of sex or gonadal status. The experimental *T*_a_, sex or gonadal status did not affect **d** the preferred cage for food consumption, but orchiectomy and the experimental *T*_a_ altered **e** the preferred cage for defecation. Two-way ANOVA: ^$$^ *p*_S_ < .01, ^$$$^ *p*_S_ < .001, ^#^ *p*_G_ < .05, ^+^ *p*_S×G_ < .05, ^++^ *p*_S×G_ < .01, and Tukey test: *p < .05, **p < .01. *Error bar* indicates SEM, *n* = 7–8 per group

The experimental *T*_a_ and sex influenced the transfer of nesting material by the mice (*p*_T_ < .001, *p*_S_ < .001, *p*_T×S_ = .013, *p*_T×G_ = .021; Fig. 4a). When the experimental *T*_a_ was at 26 °C, all mice preferred to transfer the nesting material to the TMN cage. When the experimental *T*_a_ was at 32 °C, female mice transferred the nesting material to this warmer cage while male mice did not transfer the nesting material at all. This trend of nest transfer to a warmer cage was similar to the temperature preference shown in Fig. 3a and was not affected by gonadal status. The “paperwork score,” reflecting nest destructions, was only affected by the experimental *T*_a_ without an effect of sex or gonadal status (*p*_T_ < .001; Fig. 4b). The “nest score,” which also takes the relative nest amount into account, was solely influenced by the experimental *T*_a_ (*p*_T_ < .001; Fig. 4c). Generally, the “nest score” negatively correlated with the experimental *T*_a_, but the correlations were only significant for sham-operated female mice (*r* = −.47, *p* = .030) and gonadectomized male mice (*r* = −.48, *p* = .016).

The preference to consume food in the TMN or the experimental cage was not affected by experimental *T*_a_, sex, or gonadal status, but there was a significant interaction between sex and gonadal status (*p*_S×G_ = .042; Fig. 4d). All mice generally consumed the food equally from both cages except at the experimental temperature of 26 °C when sham-operated male mice preferred food from the 26 °C experimental cage while the gonadectomized male mice preferred food from the TMN cage. The position where mice preferred to defecate was dependent on the experimental *T*_a_ and the interactions between gonadal status and the other factors (*p*_T_ < .001, *p*_T×G_ < .001, *p*_S×G_ = .002; Fig. 4e). When the experimental *T*_a_ was set at 26 °C, most mice predominantly defecated in the 26 °C cage, except gonadectomized male mice that had no preference to defecate in either cage. When the *T*_a_ was equal in both cages at 29 °C, only sham-operated male mice preferred the experimental cage while the others defecated equally in both cages. When the experimental *T*_a_ was at 32 °C, sham-operated female mice defecated more in the TMN cage, while mice of the other groups showed no preference. Interestingly, when correlating the nest-building pattern (the cage with a higher nest score) with the defecating pattern, it was evident that while all other mice defecated in the cage without the nest, gonadectomized male mice defecated more in the cage where they had built their nest.

## Discussion

The data presented in this paper demonstrate that female mice prefer a warmer environment than male mice, in agreement with previous studies [10, 21]. This article is the first to show that this sex difference in thermal preference in adult mice is not altered by GDX, suggesting that gonadal hormones are not the main driver of this sex difference in adult mice.

The preference to a higher *T*_a_ of female mice could be explained by many other mechanisms involving a complex circuit that controls whole body thermal and energy homeostasis. The differences in body compositions may affect thermal preferences. We found that female C57BL/6J mice had a higher BSA to body mass ratio than male mice of the same age. Therefore, the female mice likely had a higher heat dissipation rate from the skin to the environment and consequently a higher energy demand than male mice which may have resulted in the preference to reside at a higher *T*_a_. Moreover, this may have resulted in a higher relative daily food intake to match the higher passive heat loss. The higher food consumption of female mice was also noted in a previous study [22].

The body composition of C57BL/6J mice is sexually dimorphic: male mice have a higher lean body mass [23] with relatively more skeletal muscle mass [24] than female mice. Because heat is a byproduct of muscular activities, the higher muscle mass in male mice could explain why male mice have generally less thermal demand compared to female mice. Paul et al. [24] found that GDX abolished the sex-specific pattern of muscle mass leading to a reduction in muscle mass in castrated male mice to a comparable amount as found in ovariectomized and sham-operated female mice. This effect of castration could also explain the higher thermal demand of castrated male mice compared to sham-operated male mice in our study, while ovariectomy did not alter the thermal preference of female mice in the active phase at *T*_a_ of 26 °C.

Control of *T*_c_ is regulated by the brain. The preoptic area of the anterior hypothalamus (POAH) is the primary site that integrates afferent signals from peripheral thermoreceptors to control the physiological responses through projections to other hypothalamic areas that modulate non-shivering thermogenesis of BAT to match the overall thermal demand [25–27]. The female gonadal steroids estradiol and progesterone differently modulate the thermoregulatory set point during the estrous cycle of mammals. A decline of *T*_c_ in the pre-ovulatory phase is related to estrogen secretion in that phase while progesterone secretion at the luteal phase increases *T*_c_ [11, 28]. Furthermore, there is evidence that estradiol and testosterone differentially affect steroid-sensitive neurons in the POAH of rats [29] and the central set point of temperature in different groups of hypothalamic neurons [12, 26]. We found that when the mice were allowed to choose between 32 and 29 °C, the overall average preferred *T*_a_ was higher for female mice than for male mice in the inactive phase, suggesting a sex-dependent regulation of the temperature set point in the mouse brain. However, since our study revealed that the removal of gonadal hormones by GDX did not affect the thermal preference of both male and female mice, we cannot conclude that the sex-dependent regulation of the temperature set point in the POAH depends on the different gonadal hormones. Nevertheless, effects of gonadal hormones outside the brain might be involved. For instance, estradiol, progesterone, or testosterone differentially stimulated or inhibited the peripheral thermoreceptors [30], and estradiol and progesterone induce while testosterone reduces the thermogenesis of BAT [31, 32].

It is highly unlikely that the lack of an effect of GDX on thermal preference found in our studies is due to the sustained presence of sex hormones after GDX. Steroid hormones, including sex hormones, generally have half-lives of 4–170 min [33]. In C57BL/6 mice, the half-lives of 17β-estradiol and testosterone are less than 1 h [34, 35]. Moreover, locomotor activities and circadian rhythms have been shown to be altered as soon as 1 week after GDX [36].

Since we did not find an effect of post-pubertal GDX on thermal preference in adult mice, it is plausible that the mechanisms underlying the sex difference in thermal preference were generated prior to puberty. Sex hormones have permanent organizational effects on brain development during two sensitive periods [37]. Perinatally, testosterone secreted from the testes masculinizes the neural circuit in male mice while androgen absence in female rodents feminizes the nervous system. During puberty, elevations of gonadal hormones due to maturation of the hypothalamic-pituitary-gonadal axis strengthen the sex-dependent behaviors. In addition, it is reported that the POAH is a sexually dimorphic region in which morphological changes develop during puberty and that exposure to estrogenic compounds at puberty disturbs the development of neuronal circuits [38, 39].

## Conclusions

In summary, we found that adult female mice preferred a warmer environment than male mice of the same age, a difference that was especially apparent in the inactive phase. However, this sex difference did not depend on the presence of gonads. The sex difference in thermal preference in adult mice might be influenced by other sex-specific pathways rather than the gonadal factors or the central set point that controls the thermal balance has already been established at pre-pubertal stages.

## Additional file

Additional file 1: Figure S1. Paperwork score shows the nesting material of each score. **Table S1.** Baseline characteristics indicate the animal data from the adaptation weeks. (PDF 147 kb)

## Abbreviations

BAT: Brown adipose tissue
BSA: Body surface area
BW: Body weight
GDX: Gonadectomy
POAH: Preoptic area of the anterior hypothalamus
*T*_a_: Ambient temperature
*T*_c_: Core body temperature
TMN: Thermoneutral
TNZ: Thermoneutral zone
TPT: Temperature preference test
