# Supplementary material for: Sex difference in thermal preference of adult mice does not depend on presence of the gonads
Source: Biol Sex Differ. 2017 Jul 11;8:24. doi: 10.1186/s13293-017-0145-7 (PMC5504804; doi:10.1186/s13293-017-0145-7)
Supplement: Additional file 1: Figure S1. — Paperwork score shows the nesting material of each score. Table S1. Baseline characteristics indicate the animal data from the adaptation weeks. (PDF 147 kb) [file 13293_2017_145_MOESM1_ESM.pdf]

Figure S1 Paperwork score

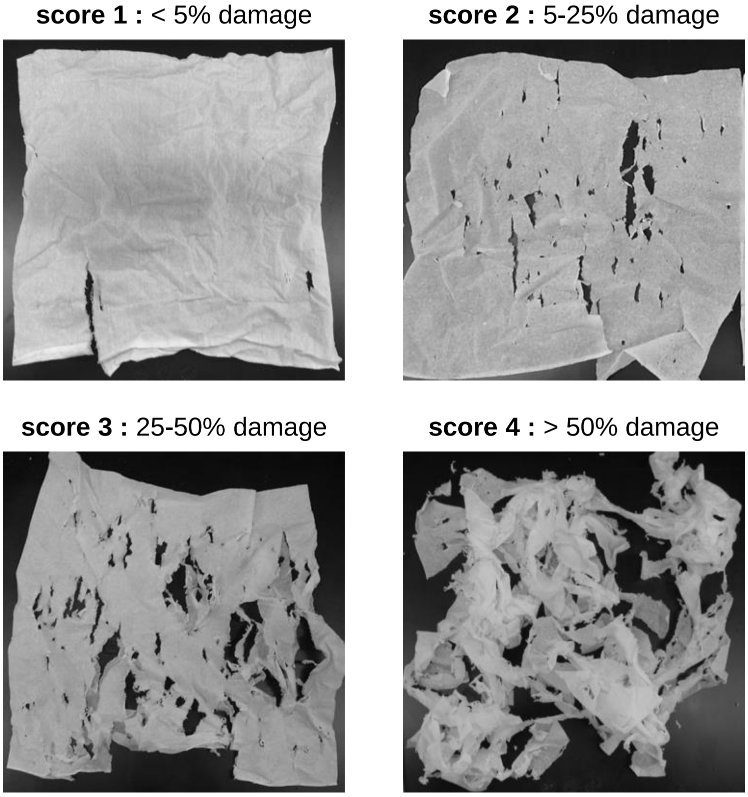

Table S1 Baseline characteristics

Characteristic	Female sham before surgery (n = 7)	Female GDX before surgery (n = 7)	Male sham before surgery (n = 7)	Male GDX before surgery (n = 8)
Initial body weight (g)	19.1 ± 0.7	19.5 ± 1.3	23.9 ± 0.8	24.0 ± 1.0
Daily body weight gain (g)	0.12 ± 0.13	0.08 ± 0.05	0.13 ± 0.05	0.19 ± 0.07
Daily food intake (g)	3.26 ± 0.44	3.10 ± 0.47	3.57 ± 0.63	3.48 ± 0.43
Food intake in TMN cage (%)	45.7 ± 9.7	47.2 ± 10.5	42.9 ± 6.9	46.8 ± 16.8
Daily fecal production (mg)	558 ± 56	576 ± 77	618 ± 86	593 ± 38
Fecal weight in TMN cage (%)	47.4 ± 10.8	44.6 ± 8.5	38.2 ± 9.0	30.9 ± 10.6
Total time in TMN cage (%)				
29 °C *vs* 26 °C day	79.1 ± 9.7	79.0 ± 9.9	70.9 ± 5.1	76.6 ± 11.1
29 °C *vs* 29 °C day	55.2 ± 26.4	61.3 ± 25.7	64.3 ± 16.5	78.6 ± 17.0
29 °C *vs* 32 °C day	46.3 ± 13.9	43.8 ± 20.6	54.9 ± 15.2	53.1 ± 22.7
Preferred temperature (˚C)				
29 °C *vs* 26 °C day	28.37 ± 0.29	28.37 ± 0.30	28.13 ± 0.15	28.30 ± 0.33
29 °C *vs* 32 °C day	30.61 ± 0.42	30.68 ± 0.62	30.35 ± 0.46	30.41 ± 0.68

All variables are described by the mean ± SD.
No difference between sham and GDX groups of the same sex. (All *p* values > .05, unpaired *t* tests)
TMN = Thermoneutral (29 ˚C), GDX = Gonadectomy
